# Understanding clinical characteristics influencing adverse outcomes of Omicron infection: a retrospective study with propensity score matching from a Fangcang hospital

**DOI:** 10.3389/fcimb.2023.1115089

**Published:** 2023-05-09

**Authors:** Yanxia Geng, Qingfang Nie, Feifei Liu, Yinghao Pei, Qiuhua Chen, Haidong Zhang, Haiqi Zhou, Jiang Zhou, Hua Jiang, Jing Xu

**Affiliations:** ^1^ Department of Intensive Care Unit, The Affiliated Hospital of Nanjing University of Chinese Medicine, Jiangsu Province Hospital of Chinese Medicine, Nanjing, Jiangsu, China; ^2^ Department of Nosocomial Infection Control, The Affiliated Hospital of Nanjing University of Chinese Medicine, Jiangsu Province Hospital of Chinese Medicine, Nanjing, Jiangsu, China

**Keywords:** COVID-19, Omicron, diarrhea, symptoms, clinical outcome

## Abstract

**Objectives:**

The epidemic of coronavirus disease 2019 (COVID-19) is causing global health concerns. The aim of this study was to evaluate influence of clinical characteristics on outcomes during the Omicron outbreak.

**Methods:**

A total of 25182 hospitalized patients were enrolled, including 39 severe patients and 25143 non-severe patients. Propensity score matching (PSM) was applied to balance the baseline characteristics. Logistic regression analysis was used to assess the risk of severe disease, as well as the risk of prolonged viral shedding time (VST) and increased length of hospital stay (LOS).

**Results:**

Before PSM, patients in the severe group were older, had higher symptom scores, and had a higher proportion of comorbidities (*p<*0.001). After PSM, there were no significant differences in age, gender, symptom score and comorbidities between severe (n=39) and non-severe (n=156) patients. Symptoms of fever (OR=6.358, 95%CI 1.748-23.119, *p*=0.005) and diarrhea (OR=6.523, 95%CI 1.061-40.110, *p*=0.043) were independent risk factors for development of severe disease. In non-severe patients, higher symptom score was associated with prolonged VST (OR=1.056, 95% CI 1.000-1.115, *p*=0.049) and LOS (OR=1.128, 95% CI 1.039-1.225, *p*=0.004); older age was associated with longer LOS (OR=1.045, 95% CI 1.007-1.084, *p*=0.020).

**Conclusion:**

The overall condition of the Shanghai Omicron epidemic was relatively mild. Potential risk factors for fever, diarrhea, and higher symptom score can help clinicians to predict clinical outcomes in COVID‐19 patients.

## Highlights

Omicron variant is a relatively mild form of SARS-CoV-2 infection.

Fever and diarrhea are two independent factors for severe Omicron infection.

Higher symptom score is related to longer VST and LOS.

## 1 Introduction

Since a cluster of cases of severe acute respiratory syndrome coronavirus 2 (SARS-CoV-2) identified in Wuhan, China in December 2019, and subsequent worldwide spread, it has caused the coronavirus disease (COVID-19) pandemic. At the time of writing, more than 761 million cases and more than 6.8 million deaths have been reported worldwide (WHO, 2023).

Currently, SARS-CoV-2 has spread globally as an Omicron variant. This variant is heavily mutated, highly contagious and has been designated as a variant of concern (VOC) by the World Health Organization (WHO) (CDC,; Gao et al., 2022). Studies have shown that Omicron variant has reduced pulmonary pathogenicity, milder spectrum of symptoms, and lower severity and mortality compared with previous VOCs (Ye et al., 2022). Here, we report a retrospective study from a Fangcang hospital during the Omicron outbreak in Shanghai, with BA.2 and BA.2.2 sublineage as the predominant variants (Zhang et al., 2022).

Fangcang hospitals are basic medical facilities transformed from large public places such as schools and exhibition centers. They can provide adequate medical services for mild and moderate patients, and prevent further spread of the virus (Chen et al., 2020). Patients who are confirmed to be positive for COVID-19 but have no symptoms or mild symptoms will be quarantined at Fangcang hospital and receive simple treatment. If the patient’s condition get worse, he or she will be transferred to appropriate hospital for further treatment. This can not only save medical resources, provide medical security for as many people as possible, but also effectively stop the spread of the virus.

Thus, the purpose of this study was to: 1) explore risk factors in the development of severe disease in Omicron-infected patients hospitalized in Fangcang hospital; 2) explore risk factors for prolonged viral shedding time (VST) and length of hospital stay (LOS) among non-severe patients using the clinical data collected from a Fangcang hospital in Shanghai.

## 2 Methods

### 2.1 Study population

This study included patients with COVID-19 infection who were admitted to Lingang Fangcang Hospital in Shanghai, China from April 6 to May 16, 2022. Lingang Fangcang Hospital was the third largest Fangcang hospital in Shanghai during the COVID-19 outbreak in 2022. Since it is a retrospective study and is part of a public health investigation, this study was approved by the Clinical Research Ethics Committee of Jiangsu Province Hospital of Chinese Medicine (2022NL-159-01), and written informed consent was waived.

For inclusion in this study it was required that:1) confirmed COVID-19 infection by the real-time reverse transcriptase-polymerase chain reaction (RT-PCR) assay of nasal or pharyngeal swab specimens for SARS-CoV-2 virus; 2) related to the Omicron outbreak in Shanghai; 3) asymptomatic or having mild symptoms; 4) age over 18 years old.

Exclusion criteria were as follows: 1) cognitive dysfunction; 2) incomplete clinical data on primary or secondary outcomes; 3) less than 24 hours during Fangcang hospital stay.

### 2.2 Treatment and outcomes

All patients were treated according to the latest COVID-19 diagnosis and treatment guidelines (trial version 9) issued by the Chinese government. Patients underwent nucleic acid RT-PCR test every day for the first three days after admission, and every other day thereafter. The basic clinical characteristics of patients, including gender, age, vaccination history, comorbidities, and symptoms (fever, sore throat, cough, expectoration, nasal congestion, running nose, fatigue, muscle ache, chest tightness, smell and taste disorder, diarrhea) were recorded. The sum of the duration of all symptoms was counted as symptom score. Patients who had received two or more doses of vaccine were considered fully vaccinated.

The primary outcome was discharge or transfer for clinical deterioration. Patients who met the following criteria were discharged: 1) normal body temperature for 3 consecutive days; 2) no obvious respiratory symptoms; 3) significant improvement of pneumonia on chest CT; 4) two consecutive negative RT-PCR test results with a 24-hour interval. On the contrary, if the patient’s condition deteriorated and met any of the following items, the patient would be transferred to a designated hospital for further treatment: 1) respiratory rate ≥ 30 times/min; 2) oxygen saturation ≤ 93% when inhaling air; 3) oxygenation index (PaO_2_/FiO_2_) ≤ 300 mmHg; 4) Shock; (5) other organ failure requiring intensive care.

Secondary outcomes were the length of VST and LOS. Since VST was not recorded in transferred patients, and the hospital stay in Fangcang hospital could not reflect the overall course of the disease, only non-severe patients were analyzed for VST and LOS.

### 2.3 Statistical analysis

We performed propensity score matching (PSM) to balance the baseline characteristics of severe and non-severe patients by 1:4 matching. SPSS software (version 18.0) was used for data analysis. Before PSM, continuous data were presented as median and interquartile ranges (IQR), and Mann Whitney U test was used for comparison between groups; categorical data were expressed as N (%), and comparison between groups was analyzed using the chi-square test. After PSM, continuous and categorical data were presented as mean ± SD and N (%), respectively. Binary logistic regression model was used to assess the effect of clinical characteristics on outcomes. Variables that were statistically significant in univariate analysis (*p* < 0.05) were included in the multivariate logistic model. All reported *p*-values were two-sided, with a significance level at 0.05.

## 3 Results

### 3.1 Baseline demographics and primary outcome

**Figure 1:**
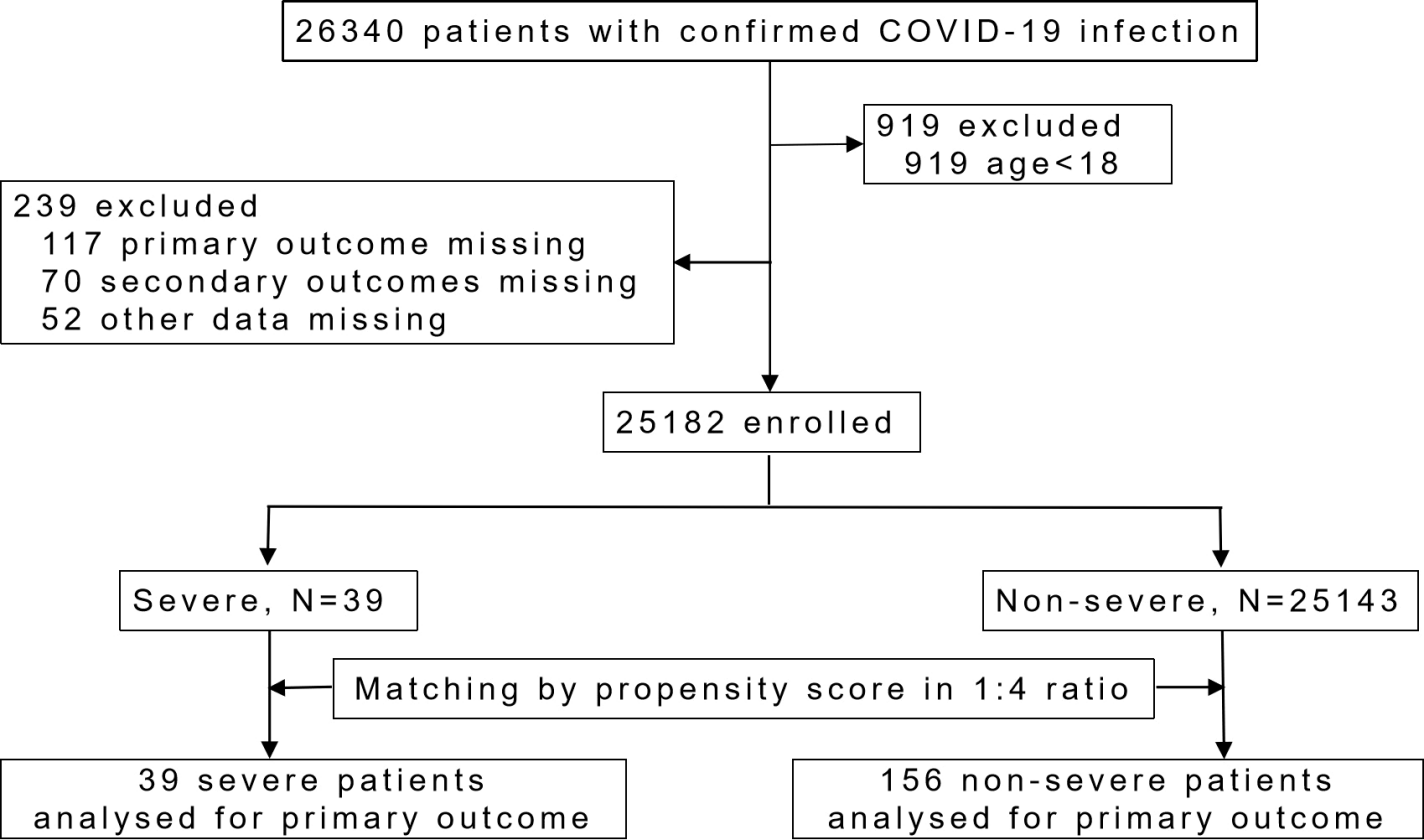
Study flow chart.

A total of 25,182 patients infected by the Omicron variant of SARS-CoV-2 were included in this study (**Figure 1**). Demographic and clinical characteristics are shown in **Supplementary Table 1**. Thirty-nine patients (0.2%) progressed to severe condition and were transferred to designated hospitals. One of them eventually died, and the rest were later recovered and discharged in the following-up. Before PSM, the severe group had significantly older age, higher symptom score, and higher proportion of comorbidities such as diabetes and hypertension than the non-severe group (*p* < 0.05, **Supplementary Table 1**). The full vaccination rate of the severe group was significantly lower than that of the non-severe group (*p* < 0.05). After PSM, there were no significant differences in age, gender, symptom score and comorbidities proportion between two groups (**Table 1**).

**Table 1:** Logistic regression analysis of risk factors associated with severe condition in patients with Omicron infection after propensity score matching.

Covariates	Severe (N=39)	Non-Severe (N=156)	Univariate analysis		Multivariate analysis	
			OR (95% CI)	*p* value	Adjusted OR (95% CI)	*p* value
**Age (x ± SD)**	58.9 ± 16.6	58.9 ± 16.5	1.000 (0.979, 1.022)	0.998		
**Gender, Male, N (%)**	22 (56.4%)	88 (56.4%)	1.000 (0.493, 2.029)	1.000		
**Symptom score (x ± SD)**	8.2 ± 6.7	7.4 ± 6.9	1.017 (0.967, 1.069)	0.515		
Symptoms, N (%)						
Fever	26 (66.7%)	13 (8.3%)	22 (9.171, 52.775)	<0.001	6.358 (1.748, 23.119)	0.005
Sore throat	24 (61.5%)	53 (34.0%)	3.109 (1.506, 6.422)	0.002	0.187 (0.035, 1.005)	0.051
Cough	26 (67.2%)	105 (67.3%)	0.971 (0.461, 2.046)	0.939		
Expectoration	25 (64.1%)	98 (62.8%)	1.057 (0.509, 2.194)	0.882		
Running nose	23 (59.0%)	49 (31.4%)	3.139 (1.525, 6.462)	0.002	0.604 (0.152, 2.390)	0.472
Nasal congestion	24 (61.5%)	50 (32.1%)	3.392 (1.639, 7.021)	0.001	0.828 (0.213, 3.218)	0.785
Muscle ache	25 (64.1%)	18 (11.5%)	13.69 (6.041, 31.025)	<0.001	3.243 (0.647, 16.250)	0.153
Fatigue	27 (69.2%)	24 (15.4%)	12.375 (5.521, 27.74)	<0.001	3.571 (0.868, 14.700)	0.078
Chest tightness	22 (14.1%)	12 (7.7%)	15.529 (6.542, 36.864)	<0.001	2.192 (0.425, 11.318)	0.349
Smell and taste disorder	20 (51.3%)	0 (0.0%)	–	–		
Diarrhea	20 (11.8%)	3 (1.9%)	53.684 (14.575, 197.73)	<0.001	6.523 (1.061, 40.110)	0.043
**With comorbidity, N (%)**	16 (41.0%)	41 (26.3%)	1.951 (0.939, 4.053)	0.073		
Diabetes	4 (10.3%)	10 (6.4%)	1.669 (0.494, 5.634)	0.410		
Hypertension	12 (30.8%)	31 (19.9%)	1.792 (0.817, 3.931)	0.145		
**Fully vaccinated, N (%)**	23 (59.0%)	125 (80.1%)	0.357 (0.168, 0.754)	0.007 ^a^	0.428 (0.143, 1.275)	0.128
**Vaccine dose, N (%)**				0.036 ^a^		
0	14 (35.9%)	30 (19.2%)	1[Reference]	–		
1	2 (5.1%)	1 (0.6%)	4.286 (0.358, 51.324)	0.251		
2	10 (25.6%)	48 (30.8%)	0.446 (0.176, 1.133)	0.090		
3	13 (33.3%)	77 (49.4%)	0.362 (0.152, 0.859)	0.021		

a The variable of “Fully vaccinated” instead of “Vaccine doses” in the univariate analysis was included in the multivariate analysis.

After PSM, we used logistic regression model to evaluate covariates that may be associated with severe Omicron infection. Results show that fever (OR=6.358, 95%CI 1.748-23.119, *p*=0.005) and diarrhea (OR=6.523, 95%CI 1.061-40.110, *p*=0.043) were risk factors for deterioration of Omicron infection. Fully vaccinated patients had meaningful *p* value in univariate regression analysis, but not in multivariate regression analysis (**Table 1**).

### 3.2 Secondary outcomes in non-severe patients

Since the majority of the population had mild Omicron infection, the aim of this study was not only to assess risk factors for developing severe disease, but also to analyze the clinical prognosis, including VST and LOS, in the non-severe population. As shown in **Tables 2**, **3**, logistic regression models were applied to analyze the risk of prolonged VST and LOS, using the 75% quartile of VST (9 days) and LOS (8 days) as cutoff values, respectively. Prolonged VST was associated with higher symptom score (OR=1.056, 95% CI 1.000-1.115, *p*=0.049) in non-severe patients (**Table 2**). Prolonged hospitalization was found to be associated with older age (OR=1.045, 95% CI 1.007-1.084, *p*=0.020), and higher symptom score (OR=1.128, 95% CI 1.039-1.225, *p*=0.004) (**Table 3**).

**Table 2:** Logistic regression analysis of risk factors for prolonged viral shedding time (VST > 9 days) in non-severe patients (N=156).

Covariates	Univariate analysis	
	OR (95% CI)	*p* value
**Age**	1.012 (0.987, 1.038)	0.340
**Male**	1.706 (0.739, 3.936)	0.211
**Symptom score**	1.056 (1.000, 1.115)	0.049
Symptoms		
Fever	0.747 (0.157, 3.562)	0.714
Sore throat	0.799 (0.337, 1.892)	0.610
Cough	1.763 (0.701, 4.433)	0.228
Expectoration	2.234 (0.892, 5.594)	0.086
Running nose	0.921 (0.387, 2.191)	0.853
Nasal congestion	1.291 (0.561, 2.971)	0.547
Muscle ache	1.231 (0.374, 4.047)	0.732
Fatigue	0.815 (0.257, 2.591)	0.729
Chest tightness	0.829 (0.172, 3.996)	0.815
Smell and taste disorder	–	–
Diarrhea	–	–
**With comorbidities**	1.259 (0.523, 3.029)	0.607
Diabetes	1.889 (0.459, 7.780)	0.379
Hypertension	1.293 (0.497, 3.364)	0.598
**Fully Vaccinated**	0.494 (0.199, 1.222)	0.127

**Table 3:** Logistic regression analysis of risk factors for prolonged hospital stay (LOS > 8 days) in non-severe patients (N=156).

Covariates	Univariate analysis		Multivariate analysis	
	OR (95% CI)	*p* value	Adjusted OR (95% CI)	*p* value
**Age**	1.033 (1.008, 1.060)	0.011	1.045 (1.007, 1.084)	0.020
**Male**	1.365 (0.641, 2.907)	0.420		
**Symptom score**	1.086 (1.029, 1.146)	0.003	1.128 (1.039, 1.225)	0.004
Symptoms				
Fever	1.481 (0.429, 5.121)	0.535		
Sore throat	1.070 (0.493, 2.321)	0.864		
Cough	1.697 (0.733, 3.930)	0.217		
Expectoration	2.627 (1.108, 6.228)	0.028	1.001 (0.304, 3.294)	0.999
Running nose	1.705 (0.791, 3.672)	0.173		
Nasal congestion	1.905 (0.889, 4.083)	0.098		
Muscle ache	1.726 (0.599, 4.973)	0.312		
Fatigue	1.400 (0.531, 3.692)	0.496		
Chest tightness	0.273 (0.034, 2.186)	0.221		
Smell and taste disorder	–	–		
Diarrhea	1.625 (0.143, 18.446)	0.695		
**With comorbidities**	1.760 (0.794, 3.906)	0.164		
Diabetes	1.412 (0.346, 5.759)	0.631		
Hypertension	1.417 (0.587, 3.424)	0.439		
**Fully vaccinated**	0.329 (0.142, 0.763)	0.010	0.559 (0.188, 1.662)	0.295

## 4 Discussion

Over the past three years, Fangcang hospital has been successfully adopted to rapidly contain several waves of SARS-CoV-2 in China, and has been adopted as a core strategy to achieve “dynamic zero”. Since the Omicron outbreak in Shanghai in March 2022, more than 100 Fangcang hospitals have been established to reduce COVID-19 cases as quickly as possible at the lowest cost, making a paramount contribution to the total reopening of Shanghai on June 1, 2022. At present, with the optimization of the COVID-19 control strategy in China, home quarantine is gradually replacing large Fangcang hospitals as the main epidemic prevention measure, and big data like this study is no longer easy to obtain.

People infected with Omicron are relatively mildly ill compared to previous COVID-19 infections (Ye et al., 2022). 25,182 patients from this cohort only shows 39 severe cases (0.2%) and 1 death (0.004%), which is significantly lower than that in Wuhan Fangcang hospitals (Sun et al., 2021). Even among non-severe patients, the average hospital stay is only 6.5 days, which is also much lower than the 10-16 days of Wuhan Fangcang hospitals (Wu S. et al., 2020; Liu et al., 2021), indicating that this wave of Omicron infection is generally milder than the original strain. The clinical characteristics of the same population are described in more detail in another paper (Ying-Hao et al., 2022).

Chronic diseases such as diabetes, and hypertension account for a higher proportion in severe and dead cases of COVID-19 (Wu and McGoogan, 2020). However, the impact of these two common comorbidities on prognosis remains controversial. Several studies have proposed hypertension and diabetes as risk factors for disease severity and mortality (Apicella et al., 2020; Guan et al., 2020; Kabia et al., 2022), and the underlying mechanisms may be related to suppression of immune function of macrophages and lymphocytes (Wu S. et al., 2020; Xu et al., 2020), as well as triggering of acute metabolic complications (Guo et al., 2020). The results of this trial do, however, align with studies showing that preexisting hypertension and diabetes have no effect on severity or mortality in COVID-19 patients (Yang et al., 2020; McFarlane et al., 2022), which may be related to our appropriate exclusion of major confounders such as age, or to Omicron’s relative mildness. In short, this finding has important public health implications for avoiding unnecessary panic caused by the constant mention of diabetes and hypertension as major risk factors for serious illness or death.

As the main finding of this study, we draw a conclusion that gastrointestinal symptom diarrhea is an independent risk factor for clinical deterioration. Evidence suggest that COVID-19 may be transmitted *via* the fecal-oral route (Xiao et al., 2020). Viruses enter cells by binding to the angiotensin- converting enzyme 2 (ACE2) receptor on the cell surface and then activating the S-protein *via* transmembrane serine protease 2 (TMPRSS2), both of which are co-expressed in intestinal epithelial cells (Dong et al., 2020). Viral particles have been detected in the small intestine of patients, mainly in MUC2-positive epithelial cells, presumably goblet cells, and in epithelial cells of crypts (Livanos et al., 2021). These studies suggest that SARS-CoV-2 virus can actively infect and replicate in the gastrointestinal tract. Disruption of intestinal epithelial cells can alter intestinal permeability, affect absorptive and secretory functions, and may explain diarrhea and other gastrointestinal manifestations (Shih and Misdraji, 2023). Meanwhile, the intestinal barrier dysfunction can trigger bacteria translocation and aggravate systemic infection and inflammation. In addition to direct viral infection, other mechanisms of gastrointestinal injury with SARS-CoV-2 may involve markedly elevated levels of inflammatory markers and cytokines (Calitri et al., 2021), as well as hypoxia due to respiratory failure, systemic coagulopathy, and right heart failure (Patel et al., 2021; Shih and Misdraji, 2023).

Studies have shown inconsistent results regarding the relationship between gastrointestinal symptoms and disease progression in COVID-19. Our study is consistent with the findings of quite a few clinical researches that patients with gastrointestinal symptoms are at higher risk for clinical deterioration than those without (Zheng et al., 2020; Zeng et al., 2022). In most of these literatures, symptoms such as anorexia, nausea, vomiting, abdominal pain, and diarrhea were grouped together as gastrointestinal symptoms. As for individual symptom, the association of diarrhea with severe disease has been variable. Approximately 10 to 20% of patients with COVID-19 develop diarrhea (Friedel and Cappell, 2023). It has been reported that late-onset diarrhea often has a poor prognosis due to COVID-19 treatments such as antibiotics, and even *Clostridioides difficile* infection (Friedel and Cappell, 2023). However, some studies did not report such findings, and even the presence of gastrointestinal symptoms is associated with lower circulating cytokine levels, reduced disease severity, and reduced mortality (Dong et al., 2020; Tabesh et al., 2022). Two recent meta-analysis also suggested no difference in mortality between patients with and without gastrointestinal symptoms (Shehab et al., 2021; Wang et al., 2022). These results indicate that the role of gastrointestinal involvement in the course of COVID-19 requires further investigation.

Fever is a common symptom of Omicron infection, and is often associated with adverse outcomes such as prolonged hospital stay (Wu S. et al., 2020; Guo et al., 2021) and development of ARDS (Wu C. et al., 2020). In our study, fever was associated with an approximately six-fold (OR=6.358) increase in the risk of severe disease, which is questionable given that fever occurred in only 8.3% of patients with non-severe disease. The possible reason for this is that some patients with mild symptoms only had fever before admission and did not report it during hospitalization, resulting in a huge discrepancy between the collected data and the real world.

The protective effect of COVID-19 vaccines has been proposed in previous studies (Hernandez-Teran et al., 2022; Kaur et al., 2022). In this research, we also found a trend toward a beneficial effect of full vaccination, but it was not significant which may be related to the relatively small sample size.

Since the main population of this study was patients with asymptomatic or mild symptoms, we also further analyzed VST and LOS in non-severe patients in an attempt to find more clues to adverse outcomes. We innovatively replaced a number of complex symptoms with a simple symptom score, and concluded that the symptom score was related to adverse outcomes such as prolonged VST, and prolonged LOS, which are theoretically reasonable and interpretable.

The limitations of this study are as follows. Firstly, it is a single-center retrospective study with a relatively small sample size after PSM, and studies with larger sample size are needed to confirm our conclusions. Second, viral nucleic acid RT-PCR test was not performed daily for each patient, resulting in a delay of VST compared with actual viral clearance time. Third, this study focused on patients’ symptoms and did not include laboratory or imaging findings and thus could not assess their prognostic role.

## 5 Conclusion

Risk factors for poor prognosis need to be identified to better understand and prevent the pandemic of COVID-19. According to our study, the overall condition of the Shanghai Omicron epidemic is relatively mild. Higher symptom score is associated with longer VST and longer hospital stay. Attention should be paid to the development of severe disease in patients with diarrhea.

## Data availability statement

The raw data supporting the conclusions of this article will be made available by the authors, without undue reservation.

## Ethics statement

This study was reviewed and approved by the Clinical Research Ethics Committee of Jiangsu Province Hospital of Chinese Medicine. Written informed consent for participation was not required for this study in accordance with the national legislation and the institutional requirements.

## Author contributions

YG drafted the manuscript. QN and FL collected the data set. YG, YP, and H-D Z analyzed the clinical data. QC and H-Q Z submitted ethics related materials. JX and HJ designed the study. JX, HJ and JZ reviewed the final manuscript. All authors approved the final manuscript.
