# Supplementary material for: Understanding clinical characteristics influencing adverse outcomes of Omicron infection: a retrospective study with propensity score matching from a Fangcang hospital
Source: Front Cell Infect Microbiol. 2023 May 9;13:1115089. doi: 10.3389/fcimb.2023.1115089 (PMC10203467; doi:10.3389/fcimb.2023.1115089)
Supplement: Supplementary file 1 [file DataSheet_1.pdf]

Supplementary Table 1, Demographic characteristics of patients with Omicron infection

Characteristics	All (N=25182)	Severe (N=39)	Non-Severe (N=25143)	*p* value
**Age, median (IQR)**	45(33,54)	59(47,73)	45(33,54)	<0.001
**Gender, Male, N(%)**	16376(65.0%)	22(56.4%)	16354(65.0%)	0.259
**Symptom score, median (IQR)**	1(0,6)	11(0,11)	1(0,6)	<0.001
**With comorbidities, N(%)**	3386(13.4%)	16(41.0%)	3370(13.4%)	<0.001
Diabetes	897(3.6%)	4(10.3%)	893(3.6%)	0.024
Hypertension	2627(10.4%)	12(30.8%)	2615(10.4%)	<0.001
**Fully vaccinated, N(%)**	20415(81.1%)	23(59.0%)	20392(81.1%)	<0.001
**Vaccine dose, N(%)**				0.003
0	3791(15.1%)	14(35.9%)	3777(15.0%)	
1	976(3.9%)	2(5.1%)	974(3.9%)	
2	8111(32.2%)	10(25.6%)	8101(32.3%)	
3	12304(48.9%)	13(33.3%)	12291(48.9%)	
